# Recent Improvement in the Long-term Survival of Breast Cancer Patients by Age and Stage in Japan

**DOI:** 10.2188/jea.JE20170103

**Published:** 2018-10-05

**Authors:** Akiyo Yoshimura, Hidemi Ito, Yoshikazu Nishino, Masakazu Hattori, Tomohiro Matsuda, Isao Miyashiro, Tomio Nakayama, Hiroji Iwata, Keitaro Matsuo, Hideo Tanaka, Yuri Ito

**Affiliations:** 1 Division of Molecular and Clinical Epidemiology, Aichi Cancer Research Institute, Nagoya, Japan; 2 Department of Breast Oncology, Aichi Cancer Center Hospital, Nagoya, Japan; 3 Department of Epidemiology, Nagoya University Graduate School of Medicine, Nagoya, Japan; 4 Division of Epidemiology & Prevention, Aichi Cancer Research Institute, Nagoya, Japan; 5 Department of Epidemiology and Public Health, Kanazawa Medical University, Ishikawa, Japan; 6 Department of Cancer Therapy Center, Fukui Prefectural Hospital, Fukui, Japan; 7 Cancer Information Services and Surveillance Division, Center for Cancer Control and Information Services, National Cancer Center, Tokyo, Japan; 8 Department of Cancer Strategy, Cancer Control Center, Osaka International Cancer Institute, Osaka, Japan; 9 Department of Cancer Epidemiology, Cancer Control Center, Osaka International Cancer Institute, Osaka, Japan

**Keywords:** breast cancer, relative survival, cancer registration, period analysis, long-term survival

## Abstract

**Background:**

Recent improvements in 5-year survival of breast cancer have been reported in Japan and other countries. Though the number of long-term breast cancer survivors has been increasing, recent improvements in 10-year survival have not been reported. Moreover, the degree of improvement according to age and disease stage remains unclear.

**Methods:**

We calculated long-term survival using data on breast cancer diagnosed from 1993 through 2006 from six prefectural population-based cancer registries in Japan. The recent increase in 10-year relative survival was assessed by comparing the results of period analysis in 2002–2006 with the results of cohort analysis in 1993–1997. We also conducted stratified analyses by age group (15–34, 35–49, 50–69, and 70–99 years) and disease stage (localized, regional, and distant).

**Results:**

A total of 63,348 patients were analysed. Ten-year relative survival improved by 2.4% (76.9% vs 79.3%) from 1993 through 2006. By age and stage, 10-year relative survival clearly improved in the age 35–49 years (+2.9%; 78.1% vs 81.0%), 50–69 years (+2.8%; 75.2% vs 78.0%) and regional disease (+3.4%; 64.9% vs 68.3%). In contrast, the degree of improvement was small in the age 15–34 years (+0.1%; 68.2% vs 68.3%), 70–99 years (+1.0%; 87.6% vs 88.6%), localized disease (+1.1%; 92.6% vs 93.7%) and distant metastasis (+0.9%; 13.8% vs 14.7%).

**Conclusions:**

These population-based cancer registry data show that 10-year relative survival improved 2.4% over this period in Japan. By age and stage, improvement in the age 15–34 years and distant metastasis was very small, which suggests the need for new therapeutic strategies in these patients.

## INTRODUCTION

Breast cancer is the most frequent female cancer in Japan, and the number of breast cancer patients continues to markedly increase. Age-standardized incidence rates using the Segi’s world population were 41.4 per 100,000 in 2003 and 63.6 per 100,000 in 2011.^1^ Prevalence was 211,500 in 2006 and is predicted to be 275,400 from 2020 to 2024.^2^ Therefore, breast cancer control will continue to be an important problem for public health policy makers, clinicians, and cancer-survivors in Japan. The analysis of a very large group of breast cancer patients from a general population allows new observations and insights, which were not clearly available at previous data scales.

In population-based studies, 5-year relative survival (RS) have been usually calculated using cohort analysis, and improvements in the 5-year RS of breast cancer have reported in Japan.^3^^,^^4^ From 1993–1996 to 2003–2005, 5-year RS improved by 4.7% (84.4% vs 89.1%). Similar improvements have been observed in European countries and the United States,^5^^,^^6^ with global surveillance of cancer survival from population-based registries showing a 5-year RS in 1995–1999 and 2005–2009 of 81.2% and 85.3% in Germany and 86.0% and 88.6% in the United States, respectively.^7^ Although most breast cancer patients survive more than 5 years, late recurrence from 5 years after diagnosis is still frequent.^8^^,^^9^ Therefore, it is essential to evaluate improvements in longer-term survival, such as 10-year survival. Furthermore, detailed analysis using the well-known prognostic factors of age and stage^3^^,^^6^ will aid both clinicians and patients in determining their long-term prognosis according to age and stage at diagnosis. Long-term follow-up would also likely help determine how long clinicians should medically follow-up their breast cancer patients. However, although long-term survival in breast cancer diagnosed before the 1990s has been evaluated,^10^^–^^12^ the improvement of long-term survival including by age and stage in more recent years has not been investigated.

To calculate 10-year RS using conventional methods (cohort approach), it requires a wait of more than 10 years after diagnosis. The results of 10-year RS using conventional methods are based on outdated data and do not match needs for clinicians and patients nowadays. Using a period approach, we could estimate more up-to date long-term survival that reflected the recent medical situation.^13^^–^^15^

Here, we aimed to report recent improvements in 10-year RS of breast cancer patients by age and stage using data from population-based cancer registries in Japan.

## METHODS

Data on a total of 70,674 female patients with invasive breast cancer (C50, International Classification of Disease for Oncology version 10) diagnosed in 1993–2006 were provided by the population-based cancer registries of six prefectures (Yamagata, Miyagi, Fukui, Niigata, Osaka, and Nagasaki) in Japan. The population covered in our study represents 13.4% of the total Japanese population and includes both urban and rural areas. Data quality in the cancer records of these prefectures is high. During 1993–2006, the percentage of Death Certificate Notified (DCN) and Death Certificate Only (DCO) cases for all sites of cancer in each prefecture were in the range of 29.1–8.1% and 21.3–4.0%, respectively. During 1995–2006, the percentage of microscopically verified cases (MV%) and mortality-to-incidence ratio (MIR) were in the range of 82.2–89.4% and 0.59–0.85, respectively. Data from these prefectures have long been used to estimate cancer survival in the national statistics.^4^ After excluding data from non-primary and non-first breast cancer patients (2,746 cases), those after initiation of therapy or with recurrence (2,622 cases), those registered by death certificate only (2,139 cases), those with uncertain diagnoses (3 cases), those aged over 99 years or under 15 years (16 cases), and those with uncertain survival time (3 cases), we finally analyzed 63,348 cases from the six prefectural cancer registries.

As patient follow-up, we used data that were followed-up for at least 5 years post-diagnosis. The cancer registries adopted linkage to the death certificate database in the prefecture to confirm the vital status of patients. Registries of Yamagata, Fukui, Osaka, and Nagasaki (partial period) additionally confirm the vital status of patients using linkage to the residential database from the death certificate. This method can complement data on patients who moved outside the prefecture where they were registered. In total, <4% of patients were lost to follow-up.

Our study was approved by the institutional review board of Osaka Medical Center for Cancer and Cardiovascular Diseases, and use of the data was approved by the cancer registries themselves. Our research project (J-CANSIS, the Japanese CANcer Survival Information for Society) has been described, including patient follow-up, in detail elsewhere.^15^

### Statistical analysis

**Figure 1.**
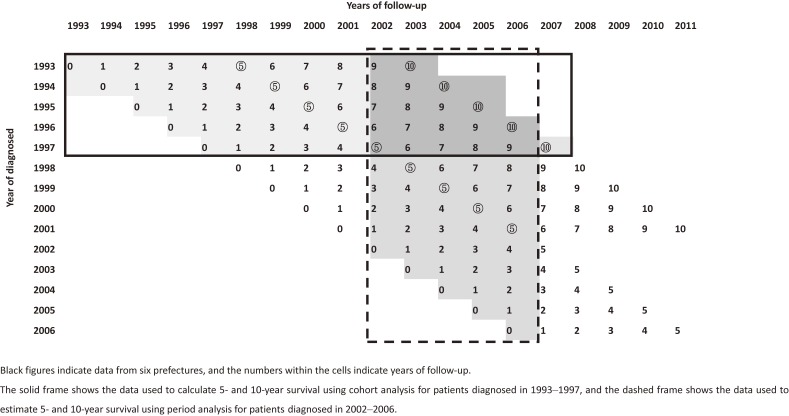
Analysed patient data

To assess recent increases in 10-year RS, period estimates of 10-year RS for the 2002–2006 periods (Figure 1, dashed frame) were calculated and compared to 10-year RS derived using classical cohort analysis for patients diagnosed in 1993–1997 (Figure 1, solid frame). In addition, we assessed recent changes in 10-year RS survival for four age groups and three disease stages. For age groups, we classified patients into those aged 34 years and under (young patients), 35–49 (premenopausal patients), 50–69 years (postmenopausal patients), and 70 years and older (elderly patients). For disease stages, we classified these by the extent of disease into localized disease (LD; UICC classification, T1-3), regional disease (RD; UICC classification, T4, N1-3b), and distant metastasis (DM; UICC classification, N3c, M1).

Relative survival is one of the standard methods to adjust for competing causes of death using population-based cancer registry data. To focus on cancer-related prognosis, relative survival rates were calculated as the ratio of the observed survival and the expected survival estimated by background mortality. We used background mortality data from the national population life tables. We then applied the maximum likelihood method as the conventional cohort analysis.^16^ Using population-based cancer registry data, the date of diagnosis (not date of initiation therapy) of cancer is applied to estimate the RS, because date of initiation therapy is not registered.

We applied period analysis to estimate 10-year RS for the 2002–2006 period. The period approach was developed to solve a problem with the cohort approach; namely, that it requires a wait of 10 years after diagnosis (Figure 1, solid frame). In contrast, the period approach allows the estimation of long-term survival using recent follow-up data (Figure 1, dashed frame).^17^^–^^19^ These statistical methods have been described in detail elsewhere.^15^

All statistical analyses were performed using the standard statistical package STATA ver. 13.1 (Stata Corp, College Station, TX, USA).

## RESULTS

The distributions of age group and disease stage according to period are summarized in Table 1. Overall, 2.7%, 30.5%, 47.7%, and 18.9% of all patients were aged 15–34, 35–49, 50–69, and 70–99 years, respectively. Age distribution has been shifted to the older age group over the period. Over half (54.7%) of all patients had a localized breast cancer, and 5.4% had distant metastasis. The proportion of patients with localized cancer has continuously increased. The major histological subtype among patients was invasive ductal carcinoma, accounting for approximately 80–85% of cases during the three diagnostic periods.

**Table 1.** Characteristics of female breast cancer patients from six prefectural population-based cancer registries

		Total		1993–1997		1998–2001		2002–2006		2002–2006 (period analysis)	
		Number	%	Number	%	Number	%	Number	%	Number	%
All patients		63,348	100.0	18,146	100.0	18,019	100.0	27,183	100.0	28,301	100.0
Age group, years	15–34	1,733	2.7	529	2.9	536	2.9	668	2.4	701	2.5
	35–49	19,365	30.5	6,636	36.5	5,520	30.6	7,209	26.5	7,522	26.6
	50–69	30,248	47.7	8,055	44.3	8,619	47.8	13,574	49.9	14,110	49.9
	70–99	12,002	18.9	2,926	16.1	3,344	18.5	5,732	21.0	5,968	21.0
Disease stage	Localized disease	34,637	54.7	9,263	51.0	9,731	54.0	15,643	57.5	16,260	57.5
	Regional disease	21,378	33.7	6,583	36.3	6,223	34.5	8,572	31.3	8,938	31.6
	Distant metastasis	3,420	5.4	994	5.5	1,005	5.6	1,421	5.2	1,483	5.2
	Unknown	3,913	6.2	1,306	7.2	1,060	5.9	1,547	5.7	1,620	5.7
Histological group	Invasive ductal carcinoma	52,710	83.2	14,637	80.6	15,067	83.6	23,006	84.6	23,960	84.7
	Mucinous carcinoma	1,739	2.7	473	2.6	495	2.8	771	2.9	802	2.8
	Invasive lobular carcinoma	1,609	2.5	392	2.2	469	2.6	748	2.8	784	2.8
	Malignant phyllodes	160	0.3	52	0.3	44	0.2	64	0.2	65	0.2
	Apocrine carcinoma	347	0.6	66	0.4	86	0.5	195	0.7	183	0.6
	Others	6,783	10.7	2,526	13.9	1,858	10.3	2,399	8.8	2,507	8.9

**Figure 2.**
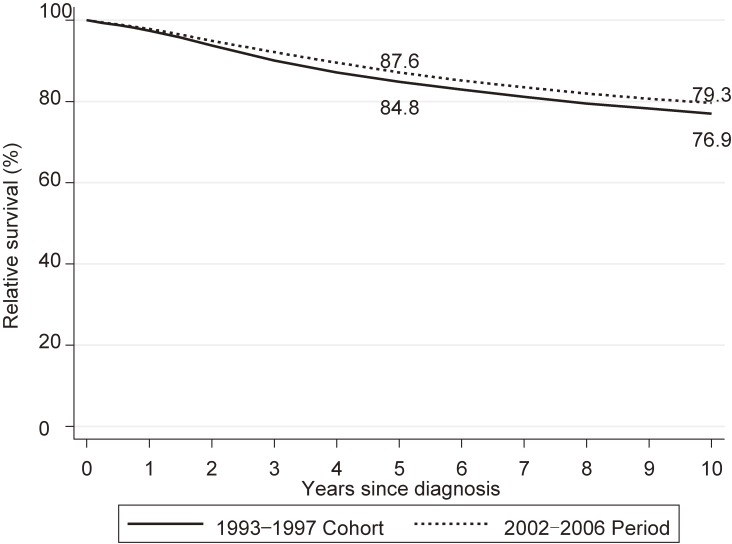
Relative survival of patients with breast cancer by period

Relative survival rates and their 95% confidence intervals are summarized in Table 2. Figure 2 shows the 10-year relative survival curve for the 2002–2006 period (dashed line) compared to that for the 1993–1997 cohort (solid line). For the 2002–2006 period, 5- and 10-year RS was 87.6% (95% confidence interval [CI], 87.1–88.0%) and 79.3% (95% CI, 78.6–79.9%), respectively. The survival rate continued to decrease over the years after diagnosis, even at 5 years after diagnosis. Survival in the 2002–2006 period was improved over that for the 1993–1997 cohort (84.8%; 95% CI, 84.2–85.4% for 5-year RS and 76.9%; 95% CI, 76.2–77.7% for 10-year RS). The improvement was 2.8% for 5-year RS and 2.4% for 10-year RS.

**Table 2.** Five- and 10-year relative survival by age and stage at diagnosis

	1993–1997 5-y RS		1993–1997 10-y RS		2002–2006 (period) 5-y RS		2002–2006 (period) 10-y RS	
	RS (%)	95% CI	RS (%)	95% CI	RS (%)	95% CI	RS (%)	95% CI
All patients	84.8	[84.2–85.4]	76.9	[76.2–77.7]	87.6	[87.1–88.0]	79.3	[78.6–79.9]
Age group, years								
15–34	78.7	[74.8–81.9]	68.2	[63.8–72.1]	81.4	[78.2–84.1]	68.3	[64.3–72.0]
35–49	86.5	[85.6–87.3]	78.1	[77.0–79.1]	90.1	[89.3–90.8]	81.0	[79.9–81.9]
50–69	82.9	[82.0–83.8]	75.2	[74.1–76.3]	85.8	[85.1–86.5]	78.0	[77.0–78.9]
70–99	88.9	[86.8–90.6]	87.6	[84.3–90.1]	90.1	[88.5–91.4]	88.6	[85.8–90.9]
Disease stage								
Localized disease	96.1	[95.6–96.6]	92.6	[91.9–93.3]	97.3	[96.9–97.6]	93.7	[93.1–94.3]
Regional disease	78.0	[76.9–79.1]	64.9	[63.5–66.2]	81.9	[81.0–82.9]	68.3	[67.0–69.5]
Distant metastasis	23.8	[21.1–26.6]	13.8	[11.6–16.2]	28.4	[25.9–30.9]	14.7	[12.5–17.0]

CI, confidence interval; RS, relative survival.

**Figure 3.**
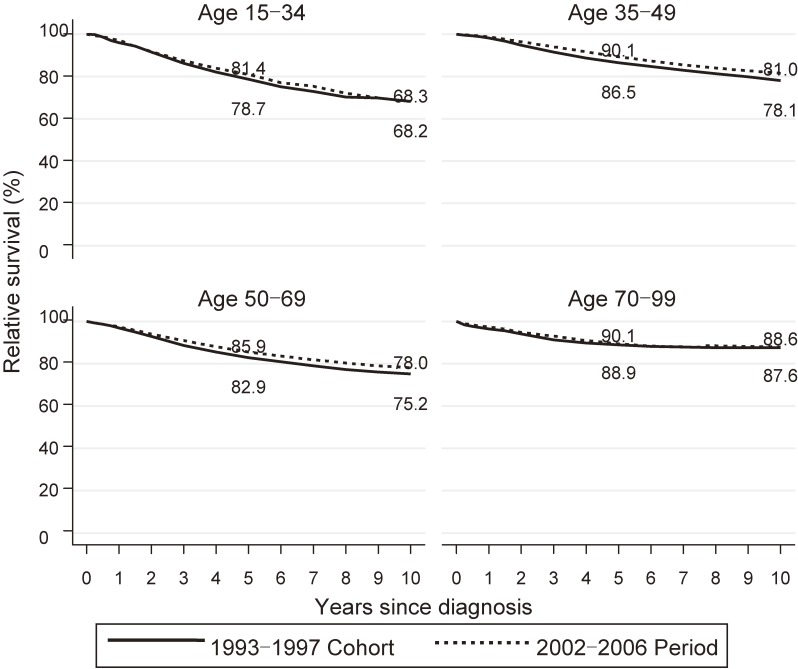
Relative survival of patients with breast cancer by age group

Figure 3 shows the 10-year RS curves for the 2002–2006 period (dashed line) compared to that for the 1993–1997 cohort (solid line) by age group. We observed an age gradient of 5-year RS, with highest RS in the oldest age group and the lowest RS in the youngest age group for both the 1993–1997 cohort analysis and the 2002–2006 period analysis. Similarly, 10-year RS rates differed among the age groups, with the highest RS in the oldest age group and the lowest RS in the youngest age group in both the 1993–1997 cohort analysis (age-specific 10-year RS rates of 68.2% [95% CI, 63.8–72.1%], 78.1% [95% CI, 77.0–79.1%], 75.2% [95% CI, 74.1–76.3%] and 87.6% [95% CI, 84.3–90.1%] for 15–34 years, 35–49 years, 50–69 years, and 70–99 years, respectively) and the 2002–2006 period analysis (age-specific 10-year RS rates of 68.3% [95% CI, 64.3–72.0%], 81.0% [95% CI, 79.9–81.9%], 78.0% 95% CI, 77.0–78.9%] and 88.6% [95% CI, 85.8–90.9%] for 15–34 years, 35–49 years, 50–69 years, and 70–99 years, respectively) (Table 2). The 2002–2006 period survival showed greater improvement than the 1993–1997 cohort survival for all age groups. The improvement in 10-year RS was large in the two middle age groups (2.9% in age 35–49 and 2.8% in age 50–69 years) compared to the youngest (0.1%) and oldest (1.0%) age groups.

**Figure 4.**
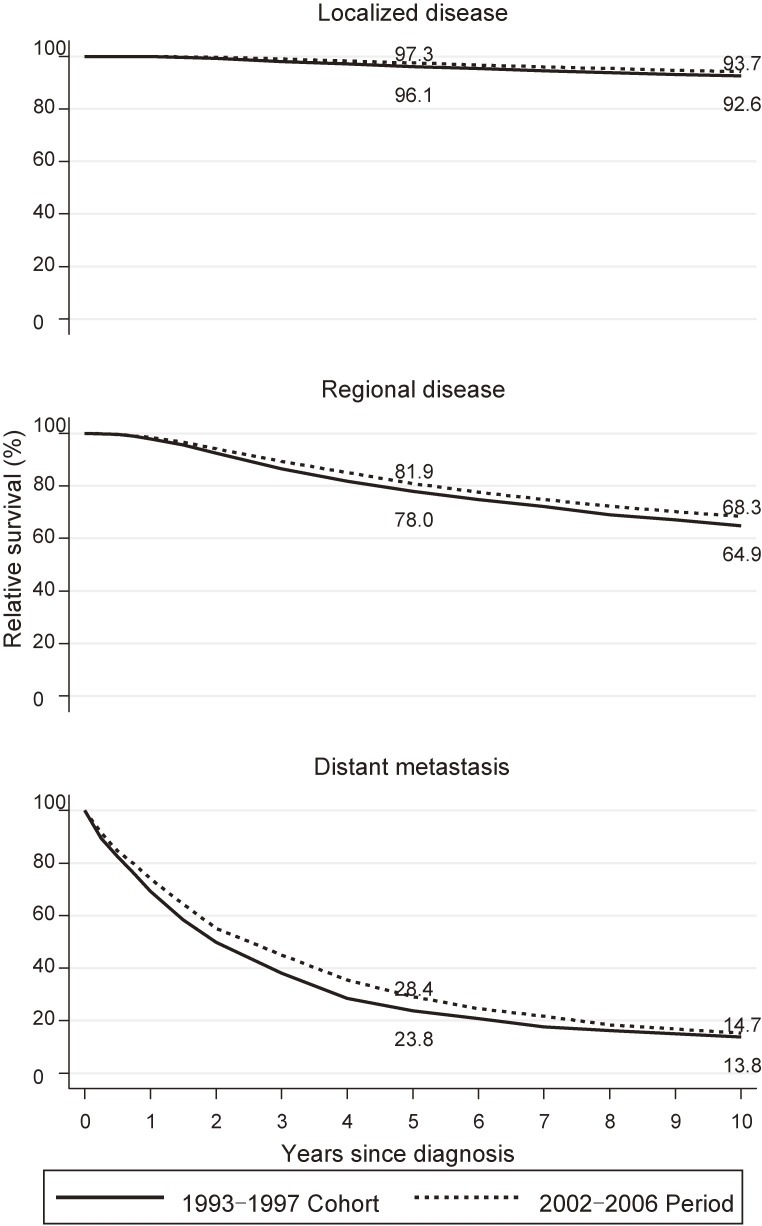
Relative survival of patients with breast cancer by disease stage

Similarly, RS for the three disease stages is shown in Figure 4. The stage gradients of 5- and 10-year RS were observed in both the 1993–1997 cohort analysis and 2002–2006 period analysis. Five-year RS was considerably improved in patients with RD (+3.9%: 78.0% [95% CI, 76.9–79.1%] to 81.9% [95% CI, 81.0–82.9%]) and DM (+4.6%: 23.8% [95% CI, 21.1–26.6%] to 28.4% [95% CI, 25.9–30.9%]) compared to patients with LD (+1.2%: 96.1% [95% CI, 95.6–96.6%] to 97.3% [95% CI, 96.9–97.6%]). We observed moderate improvement in 10-year RS among patients with RD (+3.4%: 64.9% [95% CI, 63.5–66.2%] to 68.3% [95% CI, 67.0–69.5%]) compared to those with LD (+1.1%: 92.6% [95% CI, 91.9–93.3%] to 93.7% [95% CI, 93.1–94.3%]) and DM (0.9%: 13.8% [95% CI, 11.6–16.2%] to 14.7% [95% CI, 12.5–17.0]).

## DISCUSSION

In this study, we evaluated the long-term relative survival of breast cancer patients using data from selected population-based cancer registries in Japan. There are not any population-based studies of recent improvements of 10-year RS by age and stage, as far as we know.

Five- and 10-year RS was improved by 2.8% and 2.4%, respectively, from 1993 to 2006. These improvements contrast with the greater improvements due to advances and the personalization of treatment reported in clinical trials of cancer treatment facilities.^20^^–^^23^ Although this discrepancy may be surprising, especially for clinicians, it may be attributable to the fact that patients with a relatively homogeneous background go to cancer treatment facilities and receive particularly advanced treatment, and that trial eligibility is generally limited to patients with good performance and no significant comorbidities. The improvements shown in clinical trials are not always reflected directly in the general population.

The improvement in 5- and 10-year RS might be primarily attributable to the introduction of screening mammography and advances in treatment. In Japan, organized screening mammography for women aged over 50 years started in 2000, and women in their 40s have been included since 2004. The proportion of women who had a mammography increased to 23.8% in 2006.^24^ In our data, the frequency of localized disease continuously increased over the observed period, from 51.0% to 57.5%. This increase might be due to the introduction of mammography screening and might have subsequently contributed to the overall improvement. This interpretation is always affected by lead-time and length biases.^25^^,^^26^ However, it has been demonstrated that screen-detected breast cancer confers additional prognostic benefit to the patient.^27^ We could not evaluate lead-time bias using data from cancer registries by assessing the trend of screen-detected cases and symptomatic cases because there we lacked information on motive (whether screen-detected or symptomatic) in our data.

In addition, advances in cancer treatment in Japan might also have played a role. Standard adjuvant therapy in the early 1990s consisted of oral fluoropyrimidines or classic cyclophosphamide + methotrexate + fluorouracil and anti-estrogenic drug.^28^^–^^30^ Subsequently, anthracycline + cyclophosphamide and taxan have been widely used since the late 1990s to early 2000s.^31^ Aromatase inhibitors were approved in 2001 and have been shown to be superior to anti-estrogen drugs in the prevention of recurrence of postmenopausal breast cancer.^32^

Breast cancer in young patients generally has a worse prognosis. One reason is that young patients are not candidates for breast cancer screening, so breast cancer in young patients is often found at an advanced stage.^33^ Indeed, we observed a lower proportion of LD in the young age group (46.5–49.7%) over the observed period, in contrast with proportion of LD in other age groups increasing (50.5–60.5%) over the period (eTable 1). A second reason is that breast cancer in young women often has poor biological and genetic features: triple negative subtype and BRCA gene mutations are common.^33^^–^^35^ Unfortunately, effective therapy directly targeting these adverse features has yet to appear in clinical settings. For these reasons, 10-year RS in young patients was lower than in other age groups in both observed periods, and the improvement over time was very small (68.2% vs 68.3%). Trends of 5-year RS of young breast cancer patients have been reported in Germany and the United States.^5^^,^^6^ However, these studies defined young patients as 15–49 years old, and did not evaluate 10-year survival. In our study, we divided younger patients into 15–34 years and 35–49 years old and assessed each group. It is important because young adult (15–35 years) breast cancer had worse prognosis than middle-adult breast cancer (35–50 years).^36^

In contrast, breast cancer in elderly patients generally has favorable biological and histological features: hormone receptor-positive and HER2/neu status-negative, lower proliferative rates, apocrine carcinoma, and mucinous carcinoma.^37^^–^^40^ On the other hand, elderly patients are often poor candidates for cytotoxic chemotherapy because of their susceptibility to serious side effects.^41^^,^^42^ Therefore, we observed that 10-year RS was better in elderly patients than in the other age groups, but improved only a slight 1.0% over time (87.6% vs 88.6%).

The prognosis of patients with LD was originally good, and RS was maintained at over 92% after diagnosis over the two periods. In patients with RD, 5- and 10-year RS considerably improved by 3.9% and 3.4%, respectively. Advances in radiotherapy and adjuvant therapy have reduced loco-regional and distant recurrence^31^^,^^43^^–^^45^ and might have contributed to the improvement of RS in patients with RD. With regard to DM, the algorithm of metastatic breast cancer treatment reported by Hortobagyi in 1998,^46^ which is widely supported in Japan, proposes the prolongation of survival. Moreover, aromatase inhibitors, which have improved survival of metastatic breast cancer,^47^ and trastuzumab, which is dramatically effective in HER2-positive breast cancer,^48^ were approved for metastatic breast cancer in 2001. These advances in metastatic breast cancer treatment might explain the improvement in 5-year RS in DM (23.8% vs 28.4%), but do not appear to have contributed to 10-year RS.

Comparison of stage-specific survival by period must take account of the effect of stage migration^49^^–^^51^; namely, a shift in classification towards more advanced stages due to the increased sensitivity provided by new diagnostic imaging modalities. This results in an improvement in prognosis without an effect on actual survival. However, in our study, the proportions of both RD and MD decreased through the periods. Further, classification of LD and RD in pathological stage by operation is not affected by stage migration. Therefore, it is unlikely that the improvement in survival was influenced by stage migration.

Our study has several strengths. First, we evaluated survival in a very large group of breast cancer patients from a general population, which accounted for 13.4% of the total Japanese population. Second, we evaluated improvements in 10-year RS by age and stage, though population-based studies usually calculate 5-year RS of cancer. Evaluation of longer-term RS is essential because long-term survivors of breast cancer increase. Third, our use of period analysis allowed us to estimate more up-to-date long-term survival that reflected the recent medical situation. Period analysis is based on recent data and matches needs for clinicians and cancer survivors.

Several limitations of our study should be carefully considered. First, population-based cancer registration is aimed at monitoring outcomes only, and does not contain detailed clinical information on prognostic factors (hormone receptor status, HER2/neu status, histological grade, and number of lymph node metastases) or treatment modalities (hormone therapy, chemotherapy, and radiotherapy), so we were unable to conduct further factorial analyses for outcomes. Additional factorial analysis requires the establishment of a linkage system between data from population-based cancer registration and more detailed clinical information. Such linkage is expected in Japan. Second, our analysis observed the differences in 5-year RS between the 2002–2006 period analysis (87.6%) and the cohort analysis (89.1%) (data not shown). It is possible that the estimation of RS using period analysis tends to be more pessimistic than cohort analysis when there is ongoing improvement in prognosis.^52^ Our 10-year RS calculated using period analysis might therefore have underestimated actual RS compared with the cohort analysis. Third, even though we used the latest available data in our analysis, the timeliness of cancer registration and patient follow-up in Japan still lags that in North American and northern European countries by 2–3 years, and our most recent data were from 2006. A new law on the Promotion of Cancer Registries took effect in 2016, and this should bring about an improvement in the quality of data, including timeliness and completeness.

In conclusion, we evaluated for the first time recent changes in 10-year RS by breast cancer age and stage using population-based data in Japan and found that the improvement in RS differed by age and stage. We suggest that new therapeutic strategies are needed to improve survival in patient groups in whom the improvement in RS to date has been small. Data from population-based cancer registries is highly useful in evaluating long-term survival, which is essential for long-term breast cancer survivors.

## APPENDIX A. SUPPLEMENTARY DATA

eTable 1. Stage distribution by age and periods
