# Supplementary material for: Recent Improvement in the Long-term Survival of Breast Cancer Patients by Age and Stage in Japan
Source: J Epidemiol. 2018 Oct 5;28(10):420–7. doi: 10.2188/jea.JE20170103 (PMC6143379; doi:10.2188/jea.JE20170103)
Supplement: Supplementary file 1 [file je-28-420-s001.pdf]

eTable 1. Stage distribution by age and periods

Age group, years	Disease stage	Total		1993–1997		1998–2001		2002–2006		2002–2006 (period analysis)	
		**Number**	**%**	**Number**	**%**	**Number**	**%**	**Number**	**%**	**Number**	**%**
**All patients**		63,348	100.0	18,146	100.0	18,019	100.0	27,183	100.0	28,301	100.0
**15–34**	**All patients**	1,733	100.0	529	100.0	536	100.0	668	100.0	701	100.0
	**Localized Disease**	830	47.9	246	46.5	252	47.0	332	49.7	346	49.4
	**Regional Disease**	692	39.9	211	39.9	217	40.5	264	39.5	276	39.4
	**Distant Metastasis**	95	5.5	31	5.9	32	6.0	32	4.8	34	4.8
	**Unknown**	116	6.7	41	7.7	35	6.5	40	6.0	45	6.4
**35–49**	**All patients**	19,365	100	6,636	100	5,520	100	7,209	100	7,522	100.0
	**Localized Disease**	10,448	54.0	3,379	50.9	2,967	53.8	4,102	56.9	4,243	56.4
	**Regional Disease**	6,896	35.6	2,491	37.5	1,997	36.2	2,408	33.4	2,537	33.7
	**Distant Metastasis**	821	4.2	302	4.6	240	4.3	279	3.9	293	3.9
	**Unknown**	1,200	6.2	464	7.0	316	5.7	420	5.8	449	6.0
**50–69**	**All patients**	30,248	100	8,055	100	8,619	100	13,574	100	14,110	100.0
	**Localized Disease**	16,367	54.1	4,067	50.5	4,561	52.9	7,739	57.0	8,060	57.1
	**Regional Disease**	10,358	34.2	2,993	37.2	3,037	35.2	4,328	31.9	4,484	31.8
	**Distant Metastasis**	1,805	6.0	463	5.7	541	6.3	801	5.9	832	5.9
	**Unknown**	1,718	5.7	532	6.6	480	5.6	706	5.2	734	5.2
**70–99**	**All patients**	12,002	100	2,926	100	3,344	100	5,732	100	5,968	100.0
	**Localized Disease**	6,992	58.3	1,571	53.7	1,951	58.3	3,470	60.5	3,611	60.5
	**Regional Disease**	3,432	28.6	888	30.3	972	29.1	1,572	27.4	1,641	27.5
	**Distant Metastasis**	699	5.8	198	6.8	192	5.7	309	5.4	324	5.4
	**Unknown**	879	7.3	269	9.2	229	6.9	381	6.7	392	6.6
